# Supplementary figures and images for: Deposition of complement regulators on the surface of Plasmodium falciparum merozoites depends on the immune status of the host
Source: PLoS Pathog. 2025 Apr 28;21(4):e1013107. doi: 10.1371/journal.ppat.1013107 (PMC12064020; doi:10.1371/journal.ppat.1013107)

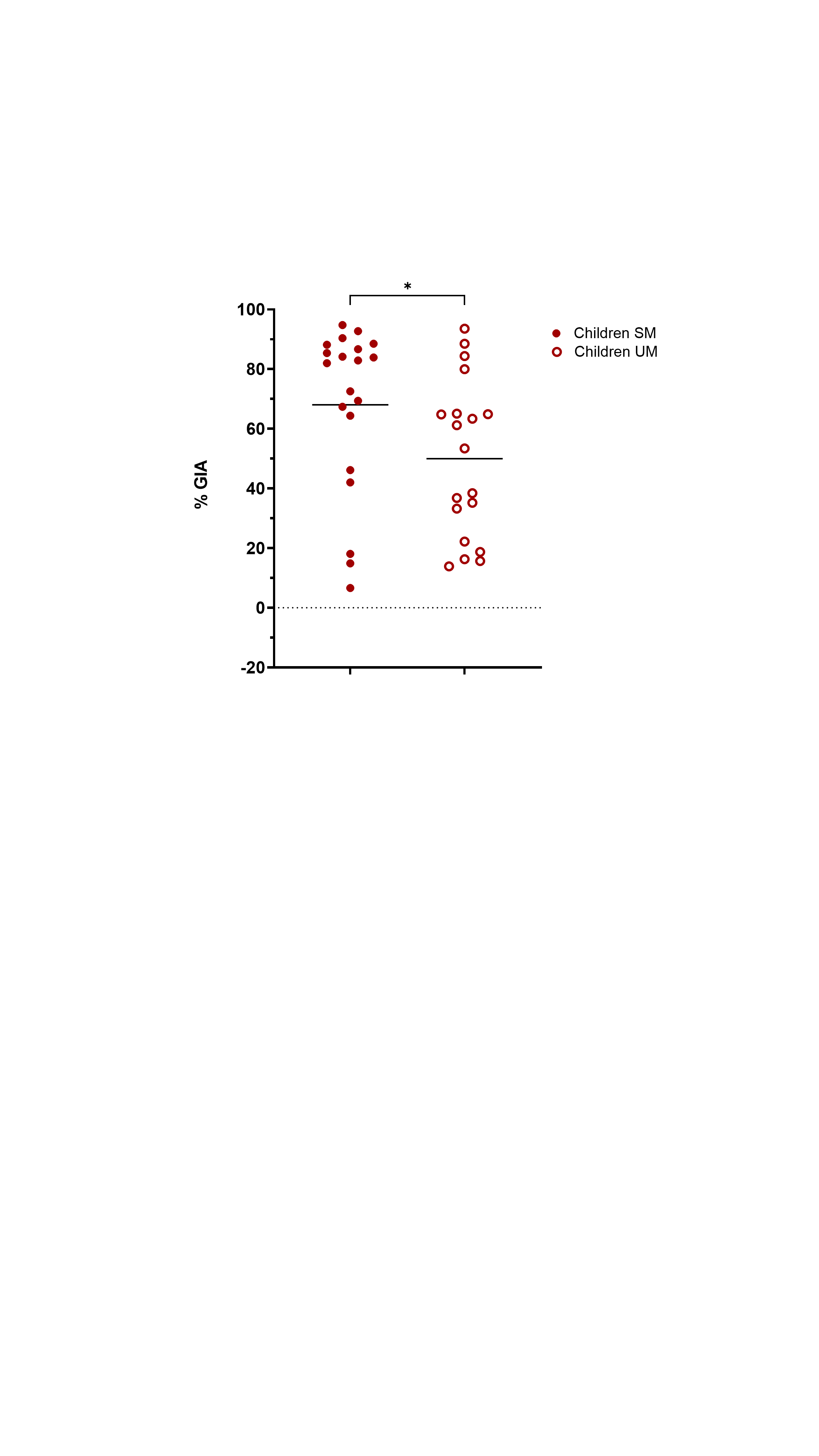

Supplement: S1 Fig — Data from Fig 1A are plotted dividing the children donors in the two clinical categories. (TIF) [file ppat.1013107.s001.tif]

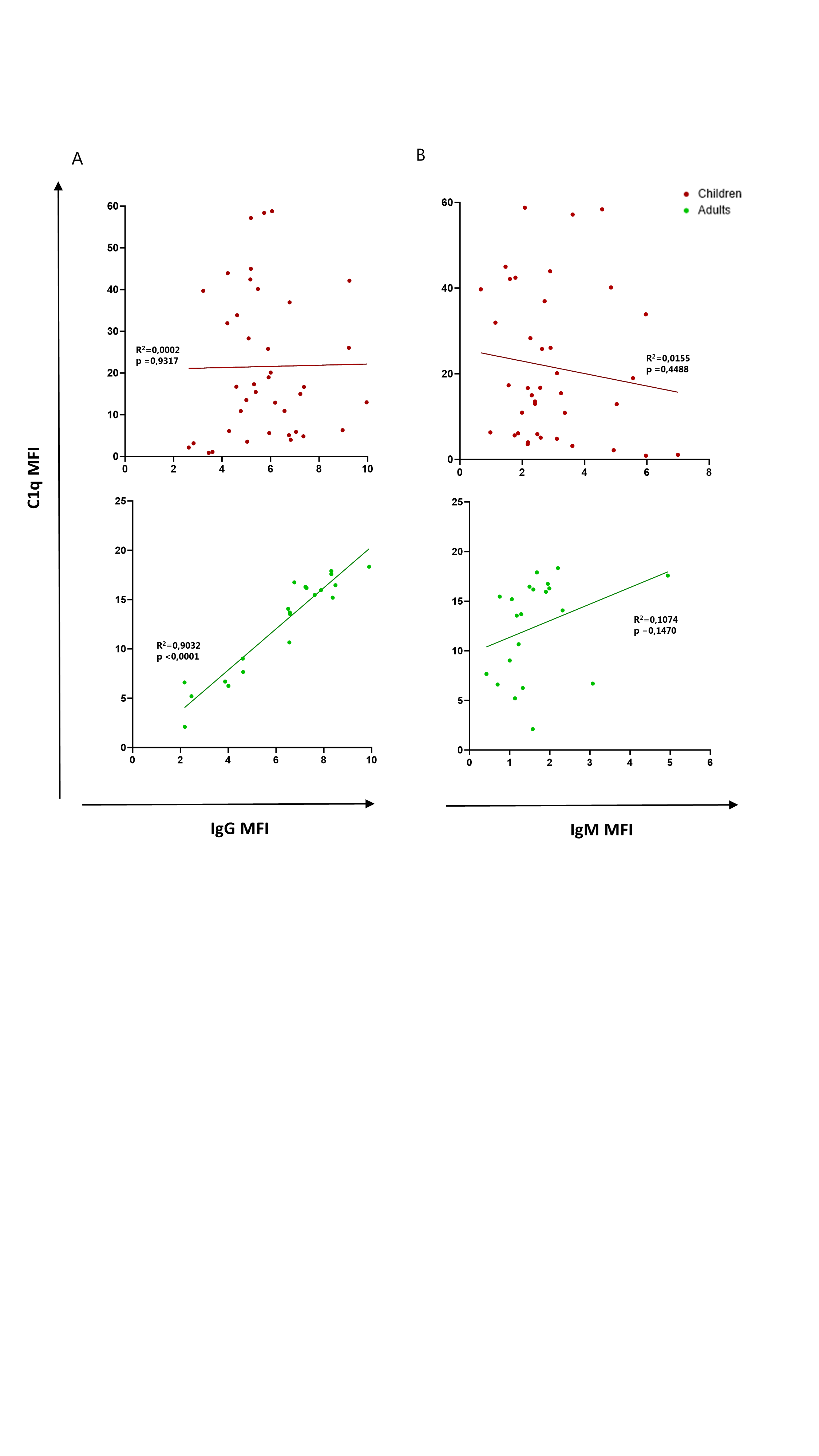

Supplement: S2 Fig — Correlation between IgG (A) or IgM (B) binding and C1q binding to merozoites after incubation with immune plasma from children (top panels, red dots) and adults (bottom panels, green dots) is measured by simple linear regression analysis. Correlation coefficients (R2) and p values are depicted in the plots. (TIF) [file ppat.1013107.s002.tif]

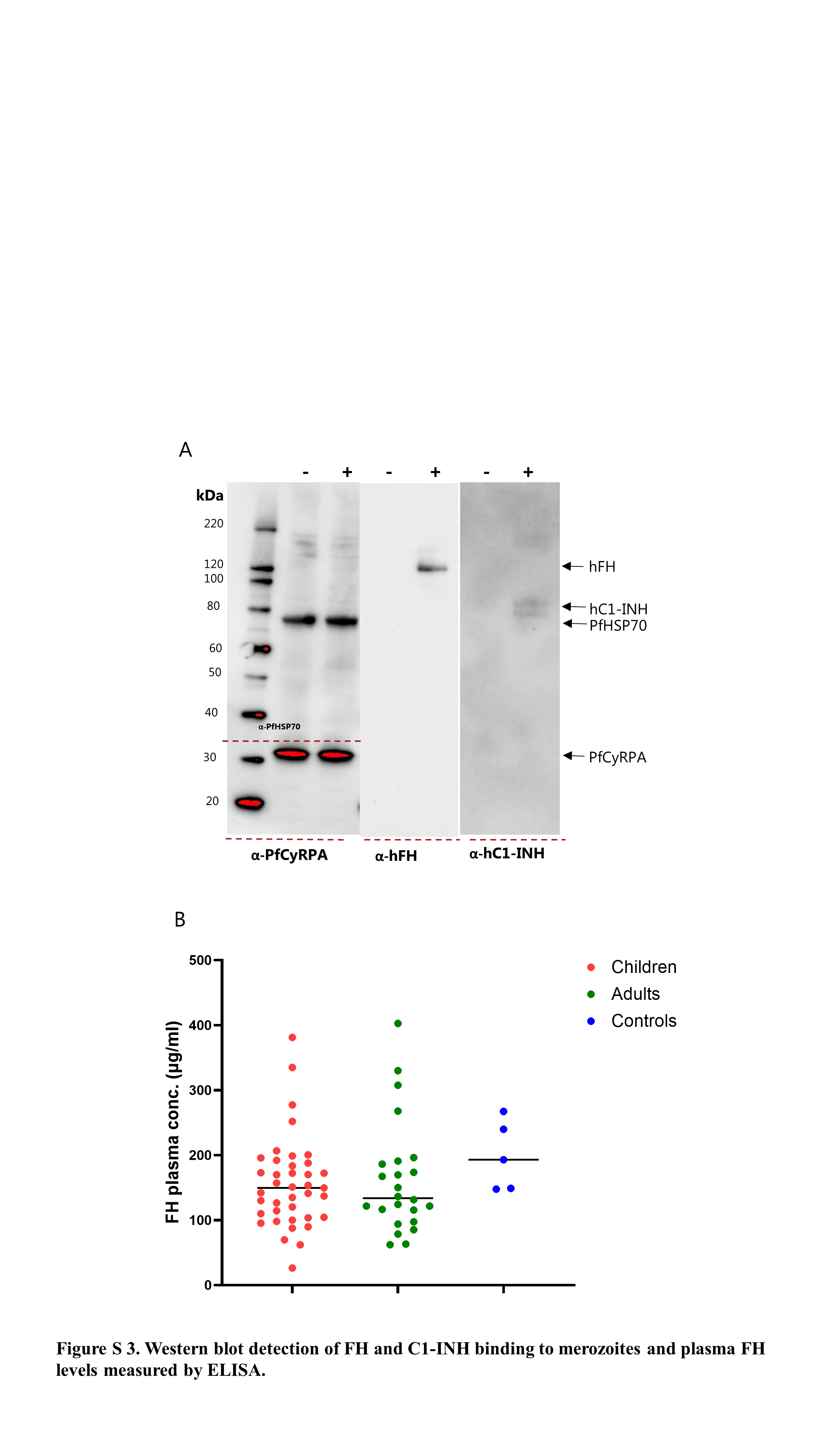

Supplement: S3 Fig — A) Western blot analysis to detect human FH, human C1-INH and the parasite control proteins PfHPS70 and PfCyRPA in protein extracts prepared from merozoites incubated with BSA control buffer (-NHS) or with NHS (+NHS). Arrows indicate protein bands corresponding to the expected size. B) Quantitative ELISA measuring the plasma levels of FH in all the immune plasma samples. FH concentration in each plasma sample is expressed as μg/mL, calculated by interpolation from a standard curve. (TIF) [file ppat.1013107.s003.tif]

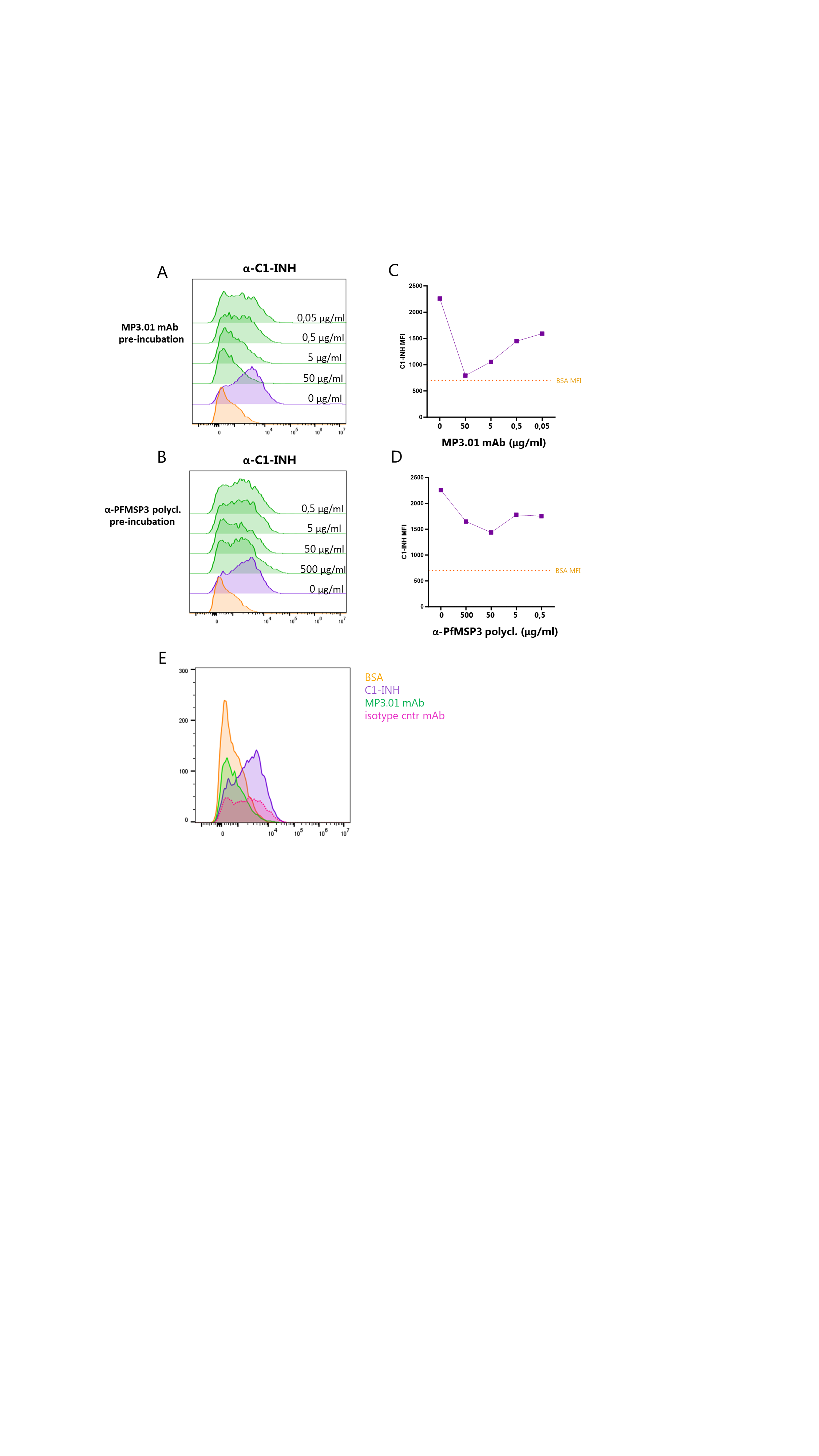

Supplement: S4 Fig — A, B) Flow cytometry analysis of human C1-INH binding to the parasite surface with and without pre-incubation of merozoites with different concentrations of MP3.01 (A) or PfMSP3 specific polyclonal IgG (B). Representative histogram plots for the staining of C1-INH are depicted. Orange curve: merozoites incubated with BSA; purple curve: merozoites incubated with C1-INH; green curves: merozoites pre-incubated with α-PfMSP3 antibodies prior to addition of C1-INH (antibody concentrations specified next to the curves). C, D) MFI values of the surface staining for human C1-INH on the merozoites surface after pre-incubation with different concentration of MP3.01 (from A) and PfMSP3 polyclonal IgG (from B) The dashed orange line corresponds to the background MFI for C1-INH obtained after staining of merozoites incubated in BSA wash buffer. E) Flow cytometry analysis of human C1-INH binding to the parasite surface after pre-incubation of merozoites with MP3.01 (50 μg/mL, green curve) or 9AD4 isotype control mAb (50 μg/mL, pink curve). Merozoites incubated with human purified C1-INH (purple curve) or BSA wash buffer (orange curve) serve as positive and negative controls, respectively. (TIF) [file ppat.1013107.s004.tif]

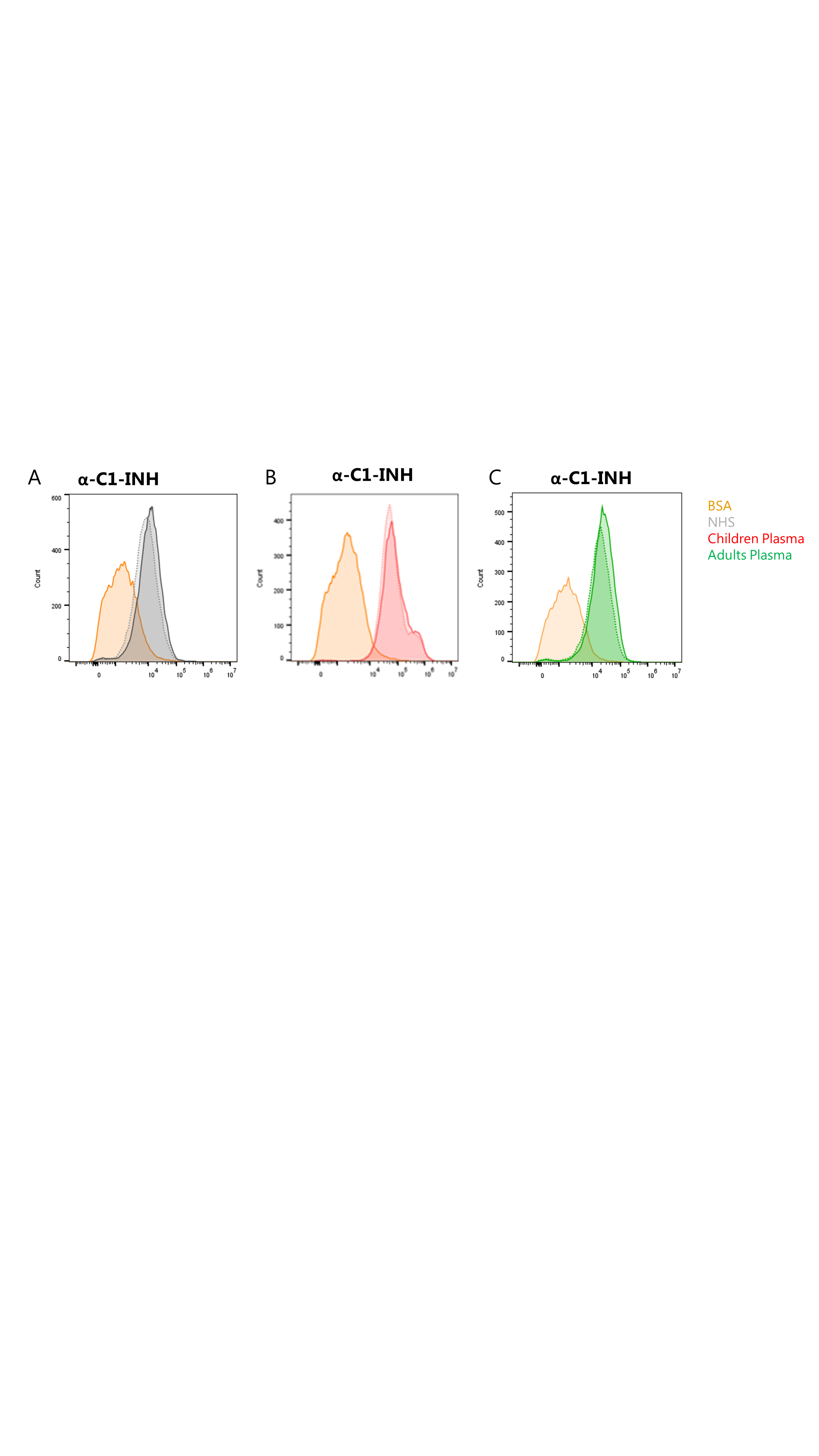

Supplement: S5 Fig — A) Flow cytometry analysis of human C1-INH binding to the parasite surface with (dotted gray line) or without (gray line) pre-incubation with 50 μg/mL of MP3.01 mAb prior to addition of 20% NHS. Representative histogram plots for the staining of C1-INH are depicted. B) Flow cytometry analysis of human C1-INH binding to the parasite surface with (dotted red line) or without (red line) pre-incubation with 50 μg/mL of MP3.01 mAb prior to addition of 20% pooled plasma from children. Representative histogram plots for the staining of C1-INH are depicted. C) Flow cytometry analysis of human C1-INH binding to the parasite surface with (dotted green line) or without (green line) pre-incubation with 50 μg/mL of MP3.01 mAb prior to addition of 20% pooled plasma from adults. Representative histogram plots for the staining of C1-INH are depicted. (TIF) [file ppat.1013107.s005.tif]

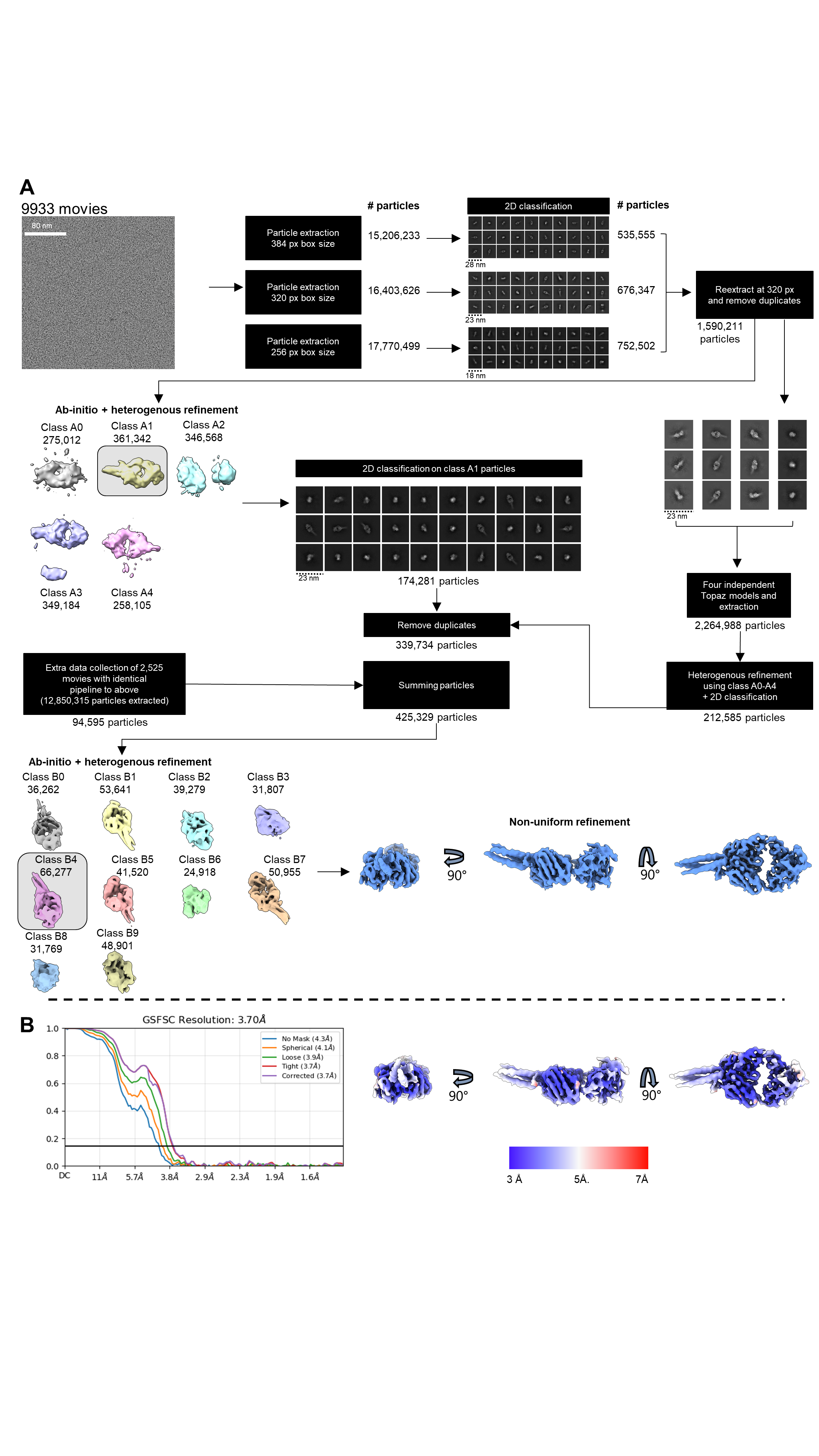

Supplement: S6 Fig — A) Workflow of generation of PfMSP3:MP3.01 electron density map. Bar on representative micrograph denotes 80 nm. Box size of 2D classes are equal to 28, 23 and 18 nm for box sizes of 384, 320, and 256 px, respectively. B) Gold Standard Fourier (GSC) shell correlation and local resolution of the electron density map. (TIF) [file ppat.1013107.s006.tif]

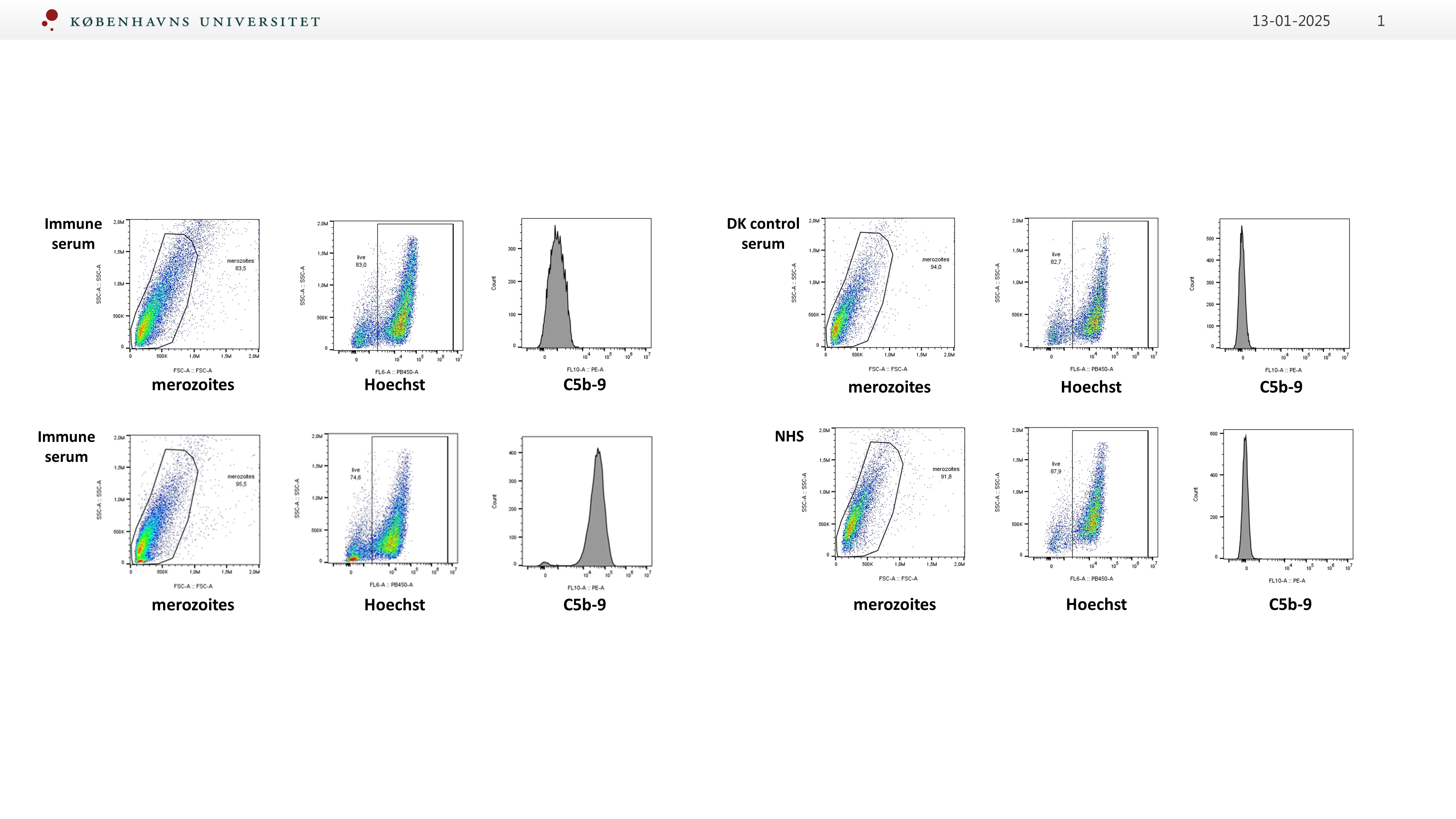

Supplement: S7 Fig — Gating for merozoites on forward versus side scatter followed by gating for Hoechst positive live cells before analysis of C5b-9 fluorescence. These plots are representative for the gating strategy of all FACS experiments. (TIF) [file ppat.1013107.s007.tif]
