# Supplementary material for: Deposition of complement regulators on the surface of Plasmodium falciparum merozoites depends on the immune status of the host
Source: PLoS Pathog. 2025 Apr 28;21(4):e1013107. doi: 10.1371/journal.ppat.1013107 (PMC12064020; doi:10.1371/journal.ppat.1013107)
Supplement: Attachment — Submitted filename: Response to Reviewers.docx [file ppat.1013107.s009.docx]

**Journal Requirements:**

**Additional Editor Comments (if provided):**

The reviewers have raised a number of important issues for you to consider. Please carefully address their concerns as you revise the manuscript.

[Note: HTML markup is below. Please do not edit.]

**Reviewers' Comments:**

Reviewer's Responses to Questions

**Part I - Summary**
Please use this section to discuss strengths/weaknesses of study, novelty/significance, general execution and scholarship.

Reviewer #1: Dear Editor,

Thank you very much for the opportunity to review the paper “Deposition of complement regulators on the surface of Plasmodium falciparum merozoites depends on the immune status of the host” by Bassi, M.R and colleagues.

In this paper the authors present a very interesting body of work investigating the role of host immune factors, specifically antibody acquisition with age, on recruitment and deposition of complement regulatory factors recently shown to be involved in parasite immune evasion to the surface of P. falciparum merozoites. Increasing evidence in the field of malaria immunity has shown that beyond direct antibody neutralisation (mediating growth inhibition) that has been shown inconsistent associations/correlation with protection from malaria, other antibody Fc-mediated functions including complement fixation on merozoite surfaces play an important role in immunity against malaria and might be more strongly correlated with protection than growth inhibition and for example greater growth inhibition is seen in the presence of antibodies plus complement compared to antibodies alone. However, recent studies have shown that P. falciparum appears to have evolved mechanisms to avoid these effector function by recruiting complement regulatory factors (Factor H (FH) and C1 esterase inhibitor (C1-INH)) to the surface of merozoites that inhibit/dampen complement activation but, unfortunately, these studies were done in the absence of naturally acquired antibodies, a major gap in our understanding of the interaction between parasites, host immunity and complement system. The authors here address this important question by investigating recruitment of these complement regulatory proteins in the presence of immune antibodies from cohort studies in Ghana and report several major findings that included important differences in immune acquisition and outcomes between children and adults including:

1. Antibodies from children induced greater growth inhibition that that of adults.
2. Antibodies from children were more effective at mediating the terminal complement cascade MAC formation compared to antibodies from adults despite similar levels of complement C1q, the initial component of the antibody dependent complement cascade
3. Factor H recruitment to the surface of merozoites was significantly lower in the presence of antibodies from children compared to adults that showed no difference with malaria naïve samples. These results suggest blocking of FH recruitment on merozoite surface by naturally acquired antibodies.
4. But for C1-1NH this was not the case with antibody samples from children shown to enhance binding of C1-1NH on merozoites suggesting they don’t compete for C1-1NH binding as for FH.
5. A monoclonal antibody against PfMSP3 inhibited/decreased binding on C1-1NH to the surface of merozoites, a fact that was not seen with naturally acquired antibodies with the authors suggesting that vaccines could be developed that are more efficient and inducing antibodies more efficient at inhibiting complement evasion by the parasites than that seen with naturally occurring antibodies.

Reviewer #2: This study emphasizes that antibodies from convalescent children recovering from malaria are notably effective in disrupting the parasite's ability to evade the immune system. These antibodies reduce the recruitment of complement regulatory proteins, particularly Factor H, which controls the alternative complement pathway. Interestingly, the study finds that while children's antibodies are more effective at hindering Factor H recruitment, antibodies from both children and adults enhance C1-INH binding (controlling the classical and lectin pathways of complement activation), The authors developed a monoclonal antibody (MP3.01) targeting PfMSP3, a parasite antigen that binds to C1-INH and aids immune evasion. This antibody blocks C1-INH binding, unlike the naturally acquired antibodies measured, which could enhance vaccine efficacy by reducing C1-INH recruitment on the merozoite surface.

Overall the manuscript was well written and clear, and I appreciate the work going into developing a mAb against PfMSP3. The study lacks an experiment that directly demonstrates whether the use of this antibody leads to improved complement activation by decreasing C1-INH recruitment. Although the structural analysis of PfMSP3 bound to the MP3.01 antibody was provided, the paper did not include functional assays to examine the effect of reduced Factor H deposition (or C1-INH deposition) on complement activation merozoite lysis. The inclusion of such experiments would significantly strengthen the paper.

**Part II – Major Issues: Key Experiments Required for Acceptance**
Please use this section to detail the key new experiments or modifications of existing experiments that should be absolutely required to validate study conclusions.

Generally, there should be no more than 3 such required experiments or major modifications for a "Major Revision" recommendation. If more than 3 experiments are necessary to validate the study conclusions, then you are encouraged to recommend "Reject".

Reviewer #1: Major comments
The major findings showing an increased inhibitory effect of FH recruitment and MAC formation by antibodies from children compared to adults while very interesting raise questions about the nature of the antibody samples in children versus adults used in this study which are my major comments/concerns in relation to the manuscript. From the cohort description provided here, lines 121 – 138, it appears very likely that these findings are an over interpretation of what could simply be explained by a huge bias of the choice of samples used and compared here and a detailed and major clarification and justification is needed to support these findings that otherwise cannot be justified. Specifically, the authors describe that plasma samples were collected from 39 children aged 1-12 years in 2015 from Hohoe in Ghana while 24 adult samples were collected from Accra Ghana in 2019.

1. From reference #26 given for the children cohort study, it appears this was a much larger study involving more than 100 children. What was the criteria used to then down-select the 39 children used here from the larger pool of 108 children that met the criteria of parasite density>2500 parasites/μl of blood? Are these 39 children part of the severe malaria (n=48) or uncomplicate malaria (n=60) group described in that study as this will greatly impact antibody responses seen? Are there any particularly important differences in the included compared to the excluded samples that could be biasing these results? A detailed explanation and comparison of different clinical and demographic details need to be given and presented of the included versus excluded samples to allay any fears of sample selection bias.

Response

We acknowledge the reviewer’s concern regarding how the children samples were down-selected and that more details about this are needed. The children plasma samples used in this study were selected among all the convalescent children with a confirmed P. falciparum infection (parasite density>2500 parasites/μl of blood) exclusively based on volume availability (plasma volume available >300 μl) and not based on disease severity or on any other clinical or demographic characteristic. Out of the 39 donors selected, 20 are from the severe malaria group and 19 from the uncomplicated malaria group, so a nearly equal distribution. Given the small size of our samples cohort, studying possible correlations between the antibody response/GIA of the plasma and disease severity is not within the scope of this paper. However, we interestingly observe a similarly variable degree of parasite growth inhibition capacity within the plasma samples from the severe malaria and the uncomplicated malaria group, rather than a direct link between clinical outcome and in vitro growth inhibition. This important information on demographic and malaria severity is now included in the “Cohort samples” paragraph of the Methods Section, in Table I and in Supplementary Figure 1.

2. No details or references have been given for the adult study samples other than that one short sentence in lines 130-131 which is rather disappointing and very frustrating when describing a cohort study and made it hard to make any reasonable judgements on the choice of these samples as was possible from the looking up the reference given for the children study sample in (1) above. The authors need to clearly outline and clarify these details as stated in the comment above for children study samples.

Response

We acknowledge that more details about the adult plasma samples are needed. Demographic details of the samples are included in table I. We have not provided references for the adult cohort, since this cohort has not been used in any other study yet. Therefore, this manuscript is the first to include data from this cohort and we cannot refer to any previous publication.

3. It is rather concerning that major conclusions are being derived comparing two groups of samples, children versus adults, that were collected from two very geographically distinct locations; Hohoe, a mostly rural area in the North Volta region (children) versus Accra, the capital and an urban area in the Southern Coastal region (adults) of Ghana. Looking at the official “Malaria Indicator Surveys” from 2016 and 2019 from the National Malaria Control Program and other bodies in Ghana, malaria prevalence (as measured in children 6-59 months) was among the highest in the Volta region at 20% and lowest nationwide (2%) in Greater Accra region in 2019 and 28% versus 5% in 2016 suggesting significant differences in malaria exposure with greater exposure to the children from Hohoe that might explain their significantly higher seroprevalence of antibodies to the difference merozoite antigens tested and in turn greater capacity as well to mediate MAC formation and inhibition of FH recruitment. It is highly likely, therefore, that these conclusions are purely a result of differences in exposure and therefore antibody acquisition by region.

Response

We agree with the reviewer that the sample collection in different geographic locations can influence features of the antibody response. Therefore, we have added comments on this in the Discussion and in the Study limitations section of the paper. Moreover, we have tried to stress that our comparison is between antibodies from recently exposed individuals (convalescent children) and in individuals who had been exposed in the past (adults). However, we would like to stress that:

- although we observed a higher seroprevalence of antibodies to a limited panel of recombinant merozoite antigens in the children (Figure 1C), the binding of total plasma IgG to live merozoites (which accounts for all the merozoite surface antigens in their native state) was similar between children and adults (Figure 2A).

- C1q deposition on the merozoites after incubation with immune plasma was similar between children and adults (Figure 2C).

These data support our idea that the superior ability to compete with the binding of FH of the children antibodies compared to the adult´s is not just a reflection of higher antibody response and/or seroprevalence.

4. Additional to (3) above these two different groups of samples were collected over two very different time periods experiencing significant changes in malaria transmission and prevalence; children in 2015 and adults in 2019. Again the “Malaria Indicator Survey” state that “The percentage of children under age 5 testing positive for malaria according to microscopy has decreased consistently over time, from 27% in 2014 to 21% in 2016 and 14% in 2019”. It would, therefore, not at all be surprising to see lower immune responses in the adult group (from 2019) compared to the children group (from 2014) even if they were to have been collected from the same geographical region, which they were not.

Response

We understand the reviewer’s concern on the different time points the children and adults samples were collected. As for for point 3, we have added comments on this in the Discussion and in the Study limitations section of the paper. Moreover, we have tried to stress that our comparison is between antibodies from recently exposed individuals (convalescent children) and individuals who had been exposed in the past (adults).

We are fully aware that we don´t have any information about how many times the individuals that participated in this study have been exposed to malaria, as this is simply not feasible.

However, we would like to stress that our data do not suggest lower immune responses in the adults compared to the children, as again IgG binding to the whole pool of merozoite antigens and C1q fixation are similar for children and adults plasma samples. The main difference we observe between the two groups is the higher IgM binding to merozoites with plasma from the children, which reflects the recent exposure of the convalescent children. This, together with the significant decline in parasite GIA of children plasma four weeks after diagnosis as compared to two weeks, suggests that the recent exposure to P. *falciparum,* rather than the location the sample where collected, determines at least some of the functional differences between children and adults antibodies.

Reviewer #2:

- Sampling issues: A major issue with interpretation throughout the manuscript is that children and adult samples were collected from different locations and years, with a four-year gap. This could affect antibody quantities and quality, as the circulating parasites may differ over time, influencing antibody pools/subtypes/and test results. For example, It’s difficulty to interpret the difference in GIA observed between the post-malaria children and adults, given that the samples were taken from different areas of Ghana, where there is substantial district-level differences in malaria risk (see PMID 38594716). The adult samples were taken from Accra, where malaria transmission may be lower than in other rural areas. Some of these differences are revealed in the antibody binding measures shown in Fig 1C, where responses to a small panel of antibodies are measured. The discussion section needs a limitation section where this addressed, and claims regarding differences between “children” and immune adults need to be tempered throughout the manuscript. (e.g., Lines 508-510).

Response

We agree with the reviewer that the sample collection in different geographic locations at different times can influence features of the antibody response. Therefore, we have added comments on this in the Discussion and in the Study limitations section of the paper. Moreover, we have tried to stress that our comparison is between antibodies from recently exposed individuals (convalescent children) and individuals who had been exposed in the past (adults). However, we would like to stress that:

However, we would like to stress that:

- although we observed a higher seroprevalence of antibodies to a limited panel of recombinant merozoite antigens for the children (Figure 1C), the binding of total IgG to live merozoites (which accounts for all the merozoite surface antigens in their native state) was similar for children and adults samples (Figure 2A).

- C1q binding to the parasite surface was similar in the presence of plasma from children and adults (Figure 2C).

This suggests that the adults do not have a lower response due to lower transmission.

- Relatedly: How are the adults known to be “clinically immune?” There is no data about whether the adults have evidence of clinical immunity, only that they live in Accra. Would change the language from “clinically immune adults” to “Ghanian adults” or something similar throughout.

Response

We agree with the reviewer comment that we present no evidence of clinical immunity in the paper. Since we do present evidence that the adults have been exposed to *P.* *falciparum* (IgG binding data by ELISA and FACS), we have changed the language to “Ghanaian adults” and “exposed adults” throughout the manuscript.

- There are a relatively small number of samples studied. 39 children (16 convalescent follow-up samples) and 24 adults. This needs to also be addressed in the discussion.

Response

We acknowledge that we need to comment on the limited samples number in the discussion. This is now mentioned in the Study Limitations section added after the Discussion.

- Figure 1A: How was the 20% cut-off for the assay defined?

Response

Values between - 20% and + 20 % are considered background in this assay because the negative control samples often give GIA % values within this interval, both when we perform the assay with plasma samples of purified Ig fraction or monoclonal antibodies. Thus, they are regarded as negative values.

- Figure 1C: The Y-axis should be labeled as "IgG" instead of "Ig" to avoid confusion that it is total Ig classes. Could the authors also plot the data from the malaria-naïve controls? And was merozoite-specific IgM measured? Could that data be included?

Response

The Y-axis label has been corrected to IgG.

The malaria-naïve controls data are not plotted in the graph as they are used to normalize the immune samples data as described in Figure Legend 1C as follows:

“IgG levels are expressed as arbitrary units (AU) obtained by dividing the OD values of the immune plasma samples by the cut-off value of each given antigen. The cut-off value was calculated for each antigen as the mean + 3SD of OD values of plasma from eight naïve plasma donors”.

Children plasma IgM responses to the same merozoite antigens used in Figure 1C have not been included in this paper, as they have been reported in a previous study from our group (reference # 25). Moreover, due to the very limited plasma volume available, we decided to prioritize the use of the samples for the FACS staining of IgM bound to native antigens on the surface of live merozoites in Figure 2B.

- Figure 2: Why didn't/couldn’t the authors separate IgG from IgM to determine which is responsible for C1q and MAC deposition in children, especially given that IgM levels are high in convalescent children? This is mentioned in the discussion (line 682-685) but would be helpful to raise when these results are first described.

Response

We have not tried to obtain IgM and IgG fractions from individual donors because we don´t have enough plasma volume for that. Therefore we have used plasma in all the experiments where we test all donors individually, including the analysis of C1q binding and MAC deposition on the merozoite surface.

We attempted IgM purification only from a pool of the top inhibitory children samples but did not succeed in obtaining any IgM. We aimed at using this IgM fraction in the GIA assay together with the children top inhibitory IgG fraction tested in Figure 1D to address the relative contribution of IgG and IgM to the high GIA of the children plasma.

- Figure 2G: What do "dk controls" refer to? Would allso include statistical comparison between children vs. controls as in 2A.

Response

The controls in figure 2G are the same malaria naïve adult individuals from Denmark used throughout this study, as stated in the figure legend. We apologize for the confusing label “dk controls” that was at the bottom of Figure 2G, which has now been removed. Statistical comparison between children and controls has been added to Figure 2G.

- Figure 3: The source and specifications of the human serum used in that experiment should be provided. Figures 3D and E could benefit from using Factor H (FH)-depleted normal human serum (NHS), and a rescue experiment adding incremental amounts of FH to FH-depleted serum could determine its impact on MAC deposition.

Response

The specifics for normal human serum and the FH-depleted human serum have now been added to the Surface staining and flow cytometry paragraph in the Methods section.

We have included the rescue experiment adding FH to FH-depleted human serum. The data are shown in Figure 3 panels F and G and comments have been added to the corresponding paragraph in the Results section.

- Correlation with IgM: In Figure 3C, it would have been helpful to examine the correlation between FH deposition and anti-Pf92 IgM levels, not just IgG.

Response

For the reasons already stated above we have not measured anti Pf92-IgM levels in this study, thus we can´t examine this correlation.

- MAC Deposition in Figure 4: Did the authors assess MAC deposition in Figure 4 the same way it was done in Figure 3 (D-E), comparing levels in purified IgG with and without C1-INH? Could that be provided?

Response

MAC deposition is not assessed in Figure 4 because there is no human serum or plasma that can act as source of MAC proteins in these experiments. The experiments described in Figure 4 are designed to confirm that the binding of C1-INH to merozoites is enhanced by immune IgG, rather than by other plasma molecules, so the merozoites are incubated with human purified C1-INH plus purified immune IgG, without any serum or plasma.

- MP3.01 Addition: In Figure 5, it would be interesting to see whether adding the MP3.01 antibody to serum from children, adults, or NHS could reduce C1-INH recruitment.

Response

We have conducted experiments where we pre-incubated merozoites with the MP3.01 mAb and observed no reduction of C1-INH recruitment from NHS or immune plasma. This is something that we aim to study further in more details, as we believe it may be due to changes in the way C1-INH binds to its specific binding partner on the surface of pathogens in the presence of immune IgG/IgM and C1q, something that is still not fully understood.

We acknowledge that this raised here by the reviewer is an important point, therefore we have added the data in Supplementary Figure 6 and commented on this point in the Study Limitations Section at the end of the Discussion.

**Part III – Minor Issues: Editorial and Data Presentation Modifications**
Please use this section for editorial suggestions as well as relatively minor modifications of existing data that would enhance clarity.

Reviewer #1: Minor comments

1. How old were the adults included in the study? Give age range details as shown for children.

Response

Demographic details of the adult donors are now provided in Table I.

2. In the text, Figures 3B and 4B are mentioned before 3A and 4A; lines 497/506 and 551/561. Swap the Figures in the actual plots.

Response

In Figure 3 and 4, panels A and B have been now swapped around as requested.

3. Lines 510-514 – so was the source of FH from the NHS or immune plasma samples? Or both? Did they measure FH in control samples too?

Response

Lines 510-514 refer to the data in Figure S2B, where we measured FH levels in all the individual immune plasma samples by quantitative ELISA. We did similarly measure FH concentration in five of the naïve control plasma samples and these data points have now been added to Supplementary Figure 3B.

We did not measure FH in NHS as we have used the same batch of a commercial NHS in all our experiments so we are not concerned of variations for that sample.

4. The suggestion that antibodies to Pf92 might compete with FH binding in parasite surface in lines 519-524 would be better shown with comparisons of levels of Factor H here with this Pf92 antibody between children and adults as in Figure 3A instead of correlation plots. It is not quite clear how different they are with the way they have presented it but looking at the data in the current format it looks like they might not be that different.

Response

We have to admit that this comment is unclear to us. We don´t understand what kind of data analysis the reviewer is suggesting instead of the correlation plot in the first three lines of the comment. Moreover, we can´t understand what “they” refer to in the last two lines of the comment.

5. The authors show in lines 524-531 and Figure 3E that Factor H interferes with MAC formation with the assay done with NHS or factor H depleted NHS. Seeing this study is looking at the role of antibodies it would have been helpful/interesting to see these two comparisons made in the presence of children versus adult antibodies as well.

Response

We have not used children and adults antibodies together with FH-depleted serum in figure 3E as here we intended to look exclusively at the activation of the alternative pathway of the complement. We have assessed MAC formation in the presence of children and adults antibodies in Figure 2G.

6. Figure 5A-B – what does the C1-NH group represent? It wasn’t clear from the text or figure legend.

Response

The C1-INH group represents merozoites incubated with human purified C1-INH in the absence of α-PfMSP3 antibodies pre-incubation, so the positive control for the C1-INH staining on the surface of merozoites. This has been now clarified in the figure legend.

7. The use of the term efficacy in regard to growth inhibition and other antibody assay endpoints is rather incorrect/misleading and should be restricted to protection/clinical outcomes for vaccine trials – e.g. lines 75, 88, 116, 612, 649, 666, 673, 701.

Response

We have substituted the term efficacy with more correct terms like neutralization capacity/ability when referring to *in vitro* antibody assays.

8. Lines 678-681 – interesting outcomes on IgM that also supported by another earlier group that deserves referencing (Boyle et al, PMID: 31579826).

Response

We thank the reviewer for suggesting this study to us that has now been added to the reference list and it corresponds to Reference # 54.

9. Format reference 55.

Response

The reference has been formatted.

10. Line 773 – “These findings”.

Response

We believe the reviewer refers to line 737 which has now been corrected.

11. Could the authors speculate on the positive correlation seen between IgG and C1q in adult samples but not in children samples despite similar levels of C1q deposition in both groups.

Response

The data mentioned here are indeed puzzling, especially since C1q and IgM do not correlate either. We speculate that the antibodies present in convalescent children may have different abilities to bind C1q, depending on the specificity of the antibody, thus we don´t see a direct link between total IgG binding and C1q binding. Moreover, the IgM the present in the children plasma samples can also bind C1q.

12. Similar to testing of GIA with purified children (pooled) IgG as shown in Figure 1D it would have been helpful to have a comparison of the same with the adult samples, for example using a pool of the highest responders from the adults group.

Response

The adults are all low responders, with 23 out of 24 donors having a GIA below the 20% cut off and only 1 donor showing GIA around 30%. Therefore, we could not analyze a pool of the highest responders

We did analyze the IgG fraction from the pool consisting of all the 24 adults and, as expected, found no parasite growth inhibition even at the highest IgG concentration tested.

13. It was not clear what results are being referred to by the huge amount of text from lines 415-427 as this reads more like a discussion of previously published work and would probably be best to be removed or significantly cut down to a sentence.

Response

This part has been slimmed and moved to the following section.

14. For all figures, check image qualities and make font sizes legible in places where they are tiny including numbers in graphs e.g. Figure 1

Response

We have improved the figures, as requested.

Reviewer #2:

 - Throughout – suggest changing language from “malaria infection” to “Plasmodium infection” or “malaria” since malaria is the disease and not what causes infection. (e.g., line 61, line 115, etc).

Response

We recognize that saying “malaria infection” crates confusion between the infectious agent and the disease, thus we have change the language as suggested throughout the manuscript.

- Line 80: might soften this language, given a recent preprint that found that Rh5.1/Matrix M might indeed have significant protection in clinical trials (<https://www.medrxiv.org/content/10.1101/2024.10.15.24315473v1>)

Response

We thank the reviewer for making us aware of this preprint. Given that this study reports a vaccine efficacy of 40-55% we feel that it is not in contradiction with our statement in line 80 about the “limited” efficacy of Rh5-based vaccines in clinical trials.

- Line 95: Define FH (first time in main text, although it is also defined in abstract)

Response

This abbreviation has now been introduced in line 95.

- In the results- lines 415-427 (*beginning with “previous work from our lab.”). This seems to be a justification for a set of experiments comparing binding, growth inhibition, and complement fixation between IgG and IgM. I was expecting data here but there are none (these data appear to be shared in the next results section.) Suggest removing these sentences here and starting the next section with a truncated version of this to help with narrative flow of the manuscript.

Response

This part has been slimmed and moved to the following section, as requested.

- Suggest removing ns from figures and putting in actual p-values

Response

ns has been substituted with p-values.

- First three paragraphs of the discussion seem redundant to the introduction? Could potentially just start with line 659?

Response

We acknowledge this redundancy and have removed lines 634 to 658 from the Discussion.

PLOS authors have the option to publish the peer review history of their article ([what does this mean?](https://journals.plos.org/plospathogens/s/editorial-and-peer-review-process#loc-peer-review-history)). If published, this will include your full peer review and any attached files.


If you choose “no”, your identity will remain anonymous but your review may still be made public.


**Do you want your identity to be public for this peer review?** For information about this choice, including consent withdrawal, please see our [Privacy Policy](https://www.plos.org/privacy-policy).

Reviewer #1: No

Reviewer #2: No

[NOTE: If reviewer comments were submitted as an attachment file, they will be attached to this email and accessible via the submission site. Please log into your account, locate the manuscript record, and check for the action link "View Attachments". If this link does not appear, there are no attachment files.]

**Figure resubmission:**

While revising your submission, please upload your figure files to the Preflight Analysis and Conversion Engine (PACE) digital diagnostic tool, https://pacev2.apexcovantage.com/. PACE helps ensure that figures meet PLOS requirements. To use PACE, you must first register as a user. Registration is free. Then, login and navigate to the UPLOAD tab, where you will find detailed instructions on how to use the tool. If you encounter any issues or have any questions when using PACE, please email PLOS at figures@plos.org. Please note that Supporting Information files do not need this step. If there are other versions of figure files still present in your submission file inventory at resubmission, please replace them with the PACE-processed versions.

Note from the authors regarding resubmission of Figure 1:

Figure 1 panel C has been modified because we realized that the previous version of the figure was missing 4 data points in the adults group for the antigens RAMA, MSRP5 and SERA9. These missing data for 4 adult donors are now included in the figure.

**Reproducibility:**

To enhance the reproducibility of your results, we recommend that authors of applicable studies deposit laboratory protocols in protocols.io, where a protocol can be assigned its own identifier (DOI) such that it can be cited independently in the future. Additionally, PLOS ONE offers an option to publish peer-reviewed clinical study protocols. Read more information on sharing protocols at https://plos.org/protocols?utm_medium=editorial-email&utm_source=authorletters&utm_campaign=protocols

*In compliance with data protection regulations, you may request that we remove your personal registration details at any time.* [*(Remove my information/details)*](https://www.editorialmanager.com/ppathogens/login.asp?a=r)*. Please contact the publication office if you have any questions.*
