# Supplementary material for: Deposition of complement regulators on the surface of Plasmodium falciparum merozoites depends on the immune status of the host
Source: PLoS Pathog. 2025 Apr 28;21(4):e1013107. doi: 10.1371/journal.ppat.1013107 (PMC12064020; doi:10.1371/journal.ppat.1013107)
Supplement: Attachment — Submitted filename: Response_to_Reviewers_auresp_2.docx [file ppat.1013107.s010.docx]

**Additional Editor Comments :**

Please address the remaining minor requests from Reviewer 1 to make some further minor clarifications in the manuscript regarding differences between the children and adult cohorts.
Additionally, in the Abstract you draw comparisons between children and adult samples - you need to make a qualification here that children and adults were taken from different endemic regions since this points was highlighted as an important limitation by both reviewers.
In the abstract, the following sentence needs revision 'In particular, we show that, while the ability to fix C1q and activate the classical pathway is similar for antibodies deriving from convalescent children and clinically immune adults, downstream complement activation shown as deposition of the membrane attack complex (MAC) is strikingly higher with antibodies developed in convalescent children.' (clarify that the subjects come from 2 different regions; higher in convalescent children compared to who?)

Response:

We acknowledge the Editors request to specify, also in the Abstract section, that the children and the adults plasma samples were collected in two different geographical areas in Ghana. We have now included this info in the Abstract stating clearly that the convalescent children samples were from Hohoe (Ghana) and the exposed adults plasma samples were from Accra (Ghana).

We have reviewed the sentence starting with “ In particular, we show…” making the comparison more clear, as requested.

**Journal Requirements:**

1) The following files are currently uploaded as file type 'Other', which are not viewable by the reviewers: (Data S1 GIA Fig 1A.xlsx,Data S2 ELISA Fig 1C.xlsx, Data S3 GIA Fig 1E.xlsx,Data S4 IgG GIA Fig 1D.xlsx, and Data S5 ELISA Fig 4C.xls). Please change the file types to 'Supporting Information' and include legends in the manuscript if you wish them to be included in review.

Response:

The files mentioned above are raw data files that we did not intend to include in the manuscript. We have uploaded them in the previous resubmission in order to comply with the journals request for submitting all the raw data behind the figures.

 

**Reviewers' Comments:**

Reviewer's Responses to Questions

**Part I - Summary**
Please use this section to discuss strengths/weaknesses of study, novelty/significance, general execution and scholarship.

Reviewer #1: Thank you for the opportunity to review the revised manuscript by Bassi, M.R. and colleagues titled "Deposition of complement regulators on the surface of Plasmodium falciparum merozoites depends on the immune status of the host"

The authors address important gaps in our understanding of how Plasmodium falciparum has evolved to evade important protective antibody Fc functions, specifically complement attack, by studying for the first time these interactions in the presence of antibodies against P. falciparum merozoites and revealing their impact on recruitment of complement proteins needed for immune evasion. They compared antibody responses from differently exposed individuals (children versus adults). Antibodies from children, but not adults, interfered Factor H recruitment (mediator of immune evasion) despite similar levels of complement C1q fixation and a monoclonal antibody to PfMSP3 blocked recruitment of C1-INH, another downregulator of the complement cascade mediating evasion, to the parasite surface suggesting a role for vaccination to induce antibodies to specific antigens that limit recruitment of certain complement proteins and in turn parasite immune evasion.

Reviewer #2: The authors have addressed the concerns raised in the prior round of review. I remain concerned about the cohort comparisons, but the authors have tempered their findings and adjusted language throughout the manuscript

**Part II – Major Issues: Key Experiments Required for Acceptance**
Please use this section to detail the key new experiments or modifications of existing experiments that should be absolutely required to validate study conclusions.

Generally, there should be no more than 3 such required experiments or major modifications for a "Major Revision" recommendation. If more than 3 experiments are necessary to validate the study conclusions, then you are encouraged to recommend "Reject".

Reviewer #1: I appreciate efforts made by the authors to address the reviewer comments and at this stage only have minor comments.

1. While I appreciate the authors adding details clarifying on the selection criteria for the 39 donor samples, could they also explicitly add the point they mention in the responses to reviewers that these samples were (randomly) selected purely based on volume availability. This point has not been added to the current corrections.

Response:

We agree with the reviewer that we should state the above more clearly in the paper. We have now added a few lines in the “Cohort Samples” paragraph of the Methods Section where we clearly state that the 39 children samples used in our study where selected based exclusively on volume availability and not on any other clinical or demographic characteristic.

2. Under the now added "study limitations" section, after the first sentence (lines 847-850), consider also adding briefly in the actual manuscript text the points they have mentioned in their responses to reviewers, point #3, stating that their comparison was between recently (children) and previously exposed individuals (adults) (also raised in response #4), and IgG to live merozoites and C1q deposition were similar in the two populations. They are important points that should be incorporated in the discussion text (not just responses to reviewers) so kindly confirm this has been captured well.

Response:

We agree with the reviewer that the above mentioned points should be added to the manuscript. We have now inserted them in the “study limitations” section after line 850, as suggested.

Reviewer #2: n/a

**Part III – Minor Issues: Editorial and Data Presentation Modifications**
Please use this section for editorial suggestions as well as relatively minor modifications of existing data that would enhance clarity.

Reviewer #1: Check throughout the manuscript for typos for example lines #19, 26, 53, 585

Response:

We apologize for the typos. We have inspected the manuscript and corrected them.

Reviewer #2: (No Response)

PLOS authors have the option to publish the peer review history of their article ([what does this mean?](https://eur02.safelinks.protection.outlook.com/?url=https%3A%2F%2Ftrack.editorialmanager.com%2FCL0%2Fhttps%3A%252F%252Fjournals.plos.org%252Fplospathogens%252Fs%252Feditorial-and-peer-review-process%2523loc-peer-review-history%2F1%2F010f0195adcee2a7-1b8a9f63-c484-4043-a3ce-2784fad5ce27-000000%2Fo_PDnstoYcL5ltnCuIc4JG5hLUOR5XuzLVTVj3PbnVE%3D203&data=05%7C02%7Cmbassi%40sund.ku.dk%7C19cc8ee7ef8b47ce95f808dd66cb5a23%7Ca3927f91cda14696af898c9f1ceffa91%7C0%7C0%7C638779745774384500%7CUnknown%7CTWFpbGZsb3d8eyJFbXB0eU1hcGkiOnRydWUsIlYiOiIwLjAuMDAwMCIsIlAiOiJXaW4zMiIsIkFOIjoiTWFpbCIsIldUIjoyfQ%3D%3D%7C0%7C%7C%7C&sdata=X4DDWxTErZb1wui%2F5RBHx%2B8JLsvtakaXsQr%2Fbh9Z%2B3E%3D&reserved=0)). If published, this will include your full peer review and any attached files.

If you choose “no”, your identity will remain anonymous but your review may still be made public.

**Do you want your identity to be public for this peer review?** For information about this choice, including consent withdrawal, please see our [Privacy Policy](https://eur02.safelinks.protection.outlook.com/?url=https%3A%2F%2Ftrack.editorialmanager.com%2FCL0%2Fhttps%3A%252F%252Fwww.plos.org%252Fprivacy-policy%2F1%2F010f0195adcee2a7-1b8a9f63-c484-4043-a3ce-2784fad5ce27-000000%2FQw1JRr8CTD3pPzVkzYS7th_kfLbZ5L4qBKuMIYiGyJk%3D203&data=05%7C02%7Cmbassi%40sund.ku.dk%7C19cc8ee7ef8b47ce95f808dd66cb5a23%7Ca3927f91cda14696af898c9f1ceffa91%7C0%7C0%7C638779745774404142%7CUnknown%7CTWFpbGZsb3d8eyJFbXB0eU1hcGkiOnRydWUsIlYiOiIwLjAuMDAwMCIsIlAiOiJXaW4zMiIsIkFOIjoiTWFpbCIsIldUIjoyfQ%3D%3D%7C0%7C%7C%7C&sdata=vKuiQRtBtmRD476L7WCvK0n0buSJW74SJIsNx2RE9vM%3D&reserved=0).

Reviewer #1: No

Reviewer #2: No

[NOTE: If reviewer comments were submitted as an attachment file, they will be attached to this email and accessible via the submission site. Please log into your account, locate the manuscript record, and check for the action link "View Attachments". If this link does not appear, there are no attachment files.]

**Figure resubmission:**

While revising your submission, please upload your figure files to the Preflight Analysis and Conversion Engine (PACE) digital diagnostic tool, <https://pacev2.apexcovantage.com/>. PACE helps ensure that figures meet PLOS requirements. To use PACE, you must first register as a user. Registration is free. Then, login and navigate to the UPLOAD tab, where you will find detailed instructions on how to use the tool. If you encounter any issues or have any questions when using PACE, please email PLOS at [figures@plos.org](mailto:figures@plos.org). Please note that Supporting Information files do not need this step. If there are other versions of figure files still present in your submission file inventory at resubmission, please replace them with the PACE-processed versions.

**Reproducibility:**

To enhance the reproducibility of your results, we recommend that authors of applicable studies deposit laboratory protocols in protocols.io, where a protocol can be assigned its own identifier (DOI) such that it can be cited independently in the future. Additionally, PLOS ONE offers an option to publish peer-reviewed clinical study protocols. Read more information on sharing protocols at <https://plos.org/protocols?utm_medium=editorial-email&utm_source=authorletters&utm_campaign=protocols>
